# New insights into the regulatory function of CYFIP1 in the context of WAVE- and FMRP-containing complexes

**DOI:** 10.1242/dmm.025809

**Published:** 2017-04-01

**Authors:** Sabiha Abekhoukh, H. Bahar Sahin, Mauro Grossi, Samantha Zongaro, Thomas Maurin, Irene Madrigal, Daniele Kazue-Sugioka, Annick Raas-Rothschild, Mohamed Doulazmi, Pilar Carrera, Andrea Stachon, Steven Scherer, Maria Rita Drula Do Nascimento, Alain Trembleau, Ignacio Arroyo, Peter Szatmari, Isabel M. Smith, Montserrat Milà, Adam C. Smith, Angela Giangrande, Isabelle Caillé, Barbara Bardoni

**Affiliations:** 1 Université Côte d'Azur, Nice, France; 2 CNRS UMR 7275, Institute of Molecular and Cellular Pharmacology, 06560 Valbonne, France; 3 CNRS Associated International Laboratory (LIA) ‘Neogenex’, 06560 Valbonne, France; 4 Institut de Génétique et de Biologie Moléculaire et Cellulaire, 67400 Illkirch, France; 5 CNRS, UMR7104, 67400 Illkirch, France; 6 Institut National de la Santé et de la Recherche Médicale, U964, 67400 Illkirch, France; 7 Université de Strasbourg, 67404 Illkirch, France; 8 Biochemistry and Molecular Genetics Department, Hospital Clinic, 08036 Barcelona, Spain; 9 Center for Biomedical Research on Rare Diseases (CIBERER), Barcelona, Spain; 10 IDIBAPS, Barcelona, Spain; 11 Instituto de Pesquisa Pelé Pequeno Principe, Curitiba 80250-060, Brazil; 12 Institute of Rare Diseases, Institute of Medical Genetics, The Chaim Sheba Medical Center, Tel Hashomer 52621, Israel; 13 Sorbonne Universités, Université Pierre et Marie Curie, Univ Paris 06, CNRS UMR8256, IBPS, Neuroscience Paris Seine, France; 14 Hospital for Sick Children, Toronto, Ontario, Canada, M5G 1X8; 15 Centre for Addiction and Mental Health, Hospital for Sick Children, Department of Psychiatry, University of Toronto, Canada, M5G 1X8; 16 Departments of Pediatrics and Psychology & Neuroscience, Dalhousie University and IWK Health Centre, Halifax, Canada, B3K 6R8; 17 Department of Laboratory Medicine and Pathobiology, Faculty of Medicine, University of Toronto andProgram in Laboratory Medicine, University Health Network, Toronto, Canada; 18 Sorbonne Paris Cité, Université Paris Diderot-Paris 7, 75013 Paris, France

**Keywords:** Fragile X, Intellectual disability, Autism, CYFIP1, BP1-BP2 deletion

## Abstract

Cytoplasmic FMRP interacting protein 1 (*CYFIP1*) is a candidate gene for intellectual disability (ID), autism, schizophrenia and epilepsy. It is a member of a family of proteins that is highly conserved during evolution, sharing high homology with its *Drosophila* homolog, dCYFIP. CYFIP1 interacts with the Fragile X mental retardation protein (FMRP, encoded by the *FMR1* gene), whose absence causes Fragile X syndrome, and with the translation initiation factor eIF4E. It is a member of the WAVE regulatory complex (WRC), thus representing a link between translational regulation and the actin cytoskeleton. Here, we present data showing a correlation between mRNA levels of *CYFIP1* and other members of the WRC. This suggests a tight regulation of the levels of the WRC members, not only by post-translational mechanisms, as previously hypothesized. Moreover, we studied the impact of loss of function of both CYFIP1 and FMRP on neuronal growth and differentiation in two animal models – fly and mouse. We show that these two proteins antagonize each other's function not only during neuromuscular junction growth in the fly but also during new neuronal differentiation in the olfactory bulb of adult mice. Mechanistically, FMRP and CYFIP1 modulate mTor signaling in an antagonistic manner, likely via independent pathways, supporting the results obtained in mouse as well as in fly at the morphological level. Collectively, our results illustrate a new model to explain the cellular roles of FMRP and CYFIP1 and the molecular significance of their interaction.

## Abstract (precis)

**Summary:** CYFIP1 and FMRP interact antagonistically during neuromuscular junction growth in the fly and during new neuron differentiation in the olfactory bulb of the adult mouse.

## INTRODUCTION

Cytoplasmic FMRP interacting protein 1 (CYFIP1) is a member of the WAVE regulatory complex (WRC) along with CYFIP2, WAVE (WAS protein family member), NAP1 (NCKAP1 or HEM1 in hematopoietic cells), ABI1 (or one of its paralogous proteins, ABI2 or NESH) and HSPC300 (also known as BRK1) (Cory and Ridley, 2002). The whole complex is per se inactive, but its function is activated by the interaction between CYFIP1/2 and Rac-GTP (Derivery et al., 2009). This interaction determines the scission of the complex into two subcomplexes: one including CYFIP1/2, NCKAP1 and ABI1 and the other one associating WAVE and HSPC. This latter subcomplex interacts with Arp2/3, triggering actin polymerization (Cory and Ridley, 2002). CYFIP1 and CYFIP2 are members of a family of proteins interacting with the Fragile X mental retardation protein (FMRP, encoded by *FMR1*) in mammals, as well as in the fly (Abekhoukh and Bardoni, 2014; Schenck et al., 2001, 2003). They have different patterns of expression during brain development (Bonaccorso et al., 2015) and while CYFIP1 interacts only with FMRP, CYFIP2 is also a partner of the two other members of the Fragile X-related protein (FXR) family, namely FXR1P and FXR2P. CYFIP1 is involved in translational regulation by interacting not only with FMRP (Schenck et al., 2001), but also with the translation initiation factor 4E (Napoli et al., 2008; Beggs et al., 2015). More recently, the subcomplex including dCYFIP (the *Drosophila* ortholog of CYFIP1/2, also known as *Sra-1*) and Kette (ortholog of NAP1) has been reported to interact with a plethora of membrane proteins, including protocadherins, Roundabout (Robo) single-pass transmembrane receptors, protocadherins, netrin receptors, neuroligins, G-protein-coupled receptors and ion channels in the fly (Chen et al., 2014; Pham et al., 2016).

While a few studies have linked the CYFIP family genes to carcinogenesis (Silva et al., 2009), a large number of studies focused on the role of these proteins both within neurons and during neuronal development (Abekhoukh and Bardoni, 2014; Bonaccorso et al., 2015) because of the functional links of these proteins to neurodevelopmental disorders such as ID, autism, schizophrenia or epilepsy (Schenck et al., 2001, 2003; Madrigal et al., 2012; Wang et al., 2015; Waltes et al., 2014; De Rubeis et al., 2013; Huang, 2016). CYFIP1 is localized in the BP1-BP2 region of human chromosome 15q11.2 (Abekhoukh and Bardoni, 2014) and deletion of this region (including the *NIPA1*, *NIPA2*, *TUBGCP5* and *WHAM* genes) leads to the Burnside-Butler (BP1-BP2 microdeletion) syndrome, characterized by developmental and language delay, neurobehavioral disturbances and psychiatric problems, autism, seizures, schizophrenia and mild dysmorphic features (Cox and Butler, 2015). Up to now, analyses of the impact of CYFIP1 on mouse behaviour have been complicated by the embryonic lethality of the *Cyfip1-*null mice, which only allows analysis of the phenotype in heterozygous animals (Pathania et al., 2014). Current findings suggest a learning deficit in *Cyfip1^+/−^* heterozygous mice, as for *Fmr1-*null mice (a model for Fragile X syndrome; Bozdagi et al., 2012).

To get more insights on the cellular role of CYFIP1, we present here new data concerning its function both in the context of WAVE- and FMRP-containing complexes. We show that reduced expression of CYFIP1 results in reduced mRNA levels of the other members of the WAVE complex in mouse neurons, as well as in blood and lymphoblastoid cell lines of patients carrying the BP1-BP2 deletion of chromosome 15q11.2. These findings suggest a regulation of the mRNA levels of WRC members other than or in addition to post-translational regulation already proposed by other studies. We observed, in mouse and fly, the antagonistic character of the FMRP-CYFIP1 interaction in the definition of some neural phenotypes, which probably occurs by modulation of the mTor pathway in an antagonistic manner that is co-regulated by both proteins. Conversely, G-quadruplex-dependent translation is only driven by FMRP.

## RESULTS

### dCYFIP and dFMR1 double knockout in *Drosophila*

**Fig. 1.**
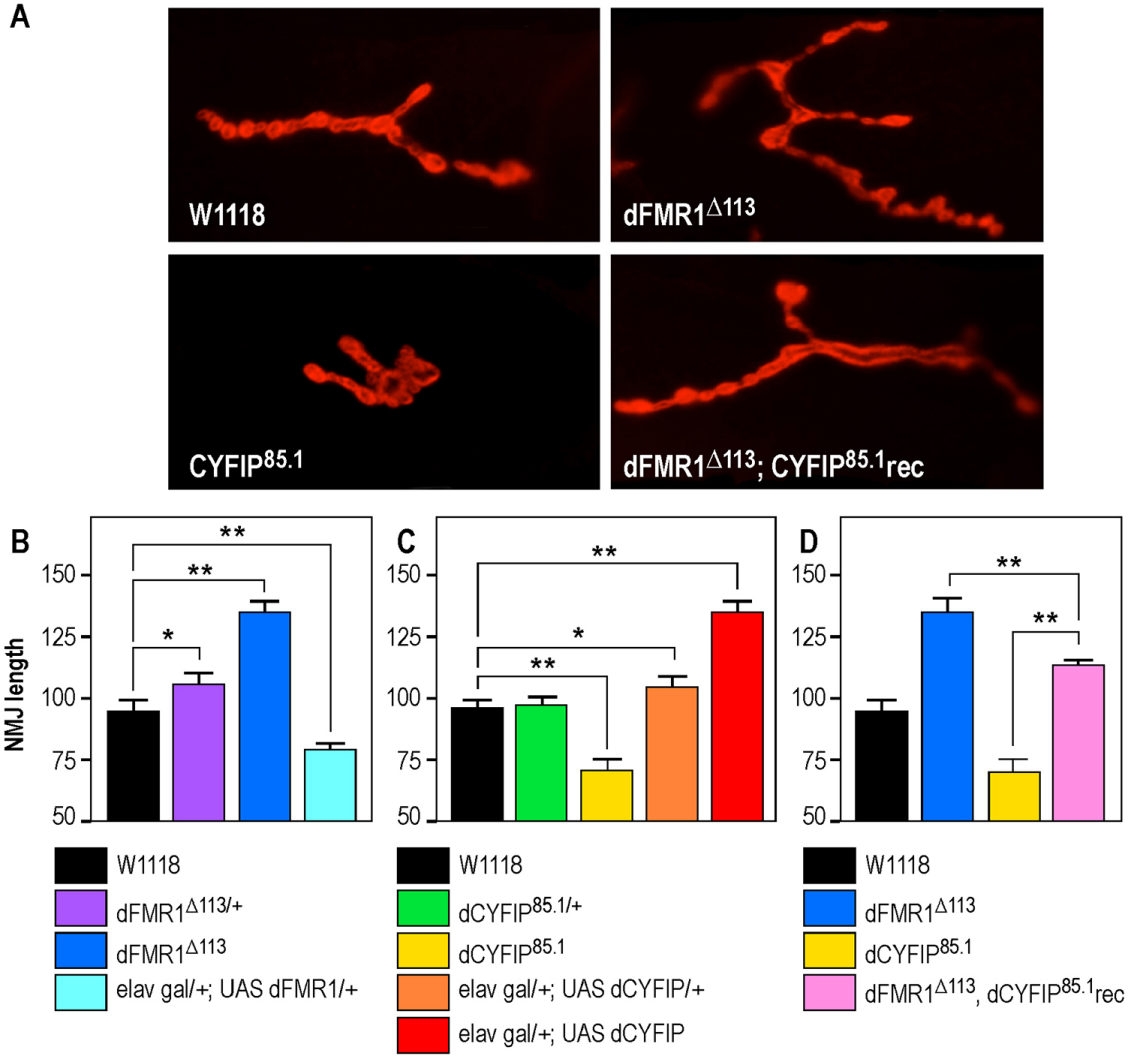
**Genetic interaction of dFMR1 and dCYFIP *in vivo*.** (A) Representative NMJs at muscle 4 labeled with a postsynaptic marker (anti-Discs large) in different *Drosophila* genotypes, as indicated. (B) NMJ quantification of depletion, knockout and one copy overexpression of *dFMR1.* (C) NMJ quantification of depletion, knockout and one- or two-copy overexpression of *dCYFIP.* (D) NMJ quantification of double *dFMR1; dCYFIP* knockout. Results are presented as the mean±s.e.m.; ANOVA and the Newman-Keuls method for *post hoc* pairwise analyses (**P<0.01; *P<0.05).

To get more insight into genetic interactions between *dCYFIP* (*Sra-1*) and *dFMR1* (*Fmr1*) (Schenck et al., 2003), we generated a double mutant for the two genes in *Drosophila* and we induced the targeted expression [gain of function (GOF)] of both genes in the larval neuromuscular junction (NMJ) system by using the presynaptic *elav-Gal4* driver. In addition, in order to have internal controls in our study, we repeated some previously published analyses (Schenck et al., 2003). NMJs provide a highly sensitive model to study synaptic plasticity and specific parameters can easily be monitored. As in previous studies (Schenck et al., 2003), we focused our analysis on NMJ length rather than area, branching level or bouton number, since this parameter is highly sensitive and useful for statistical analysis. We confirmed that dFMR1 represses synapse growth by comparing the NMJ length of wild-type larvae (*W^1118^*; Fig. 1A-D) with that of heterozygous larvae (*dFMR1^Δ113^*^/+^; Fig. 1B), that is just slightly longer (*P*=0.03), and with that of homozygous larvae (*dFMR1^Δ113^*) where NMJs are even longer (*P*<0.01) (Fig. 1A,B). Conversely, overexpression of one copy of dFMR1 (*elav gal/+; UAS-dFMR1/+*) leads to extremely short NMJs (*P*=0.01) (Fig. 1B), confirming previous observations that dFMR1 represses synaptic growth (Schenck et al., 2003). A half dose of dCYFIP via its genetic deletion (*dCYFIP^85.1/+^*) does not have an impact on NMJ length (Fig. 1C) when compared with the wild-type condition (*W^1118^*). But the total loss of dCYFIP (*dCYFIP^85.1^*) caused very short NMJs (*P*<0.01) (Fig. 1A,C). In contrast, overexpression of one copy (*elav gal/+; UAS-CYFIP/+*) had a small but significant effect (*P*<0.05) (Fig. 1C), while the overexpression of two copies of this gene (*elav gal/+; UAS-CYFIP*) induced significant overgrowth of the NMJs (*P*<0.01) (Fig. 1C). We confirmed that dCYFIP promotes synapse growth, behaving in an opposing way to dFMR1. In order to dissect the epistatic interaction between these two proteins, we explored the NMJ phenotype of transheterozygotes expressing just one copy of each gene (*dCYFIP^85.1/+^/dFMR1 ^Δ113/+^*; Fig. S1A) and we observed that this phenotype is not statistically different from that of each heterozygote (*dCYFIP^85.1/+^* and *dFMR1 ^Δ113/+^*, respectively) (Fig. S1A). In contrast, recombinant double homozygous mutant animals (*dCYFIP^85.1^; dFMR1^Δ113^* rec) (Fig. 1A,D) have NMJs significantly different from *dFMR1* and *dCYFIP* single mutants (Fig. 1D) (*P*<0.01 in both cases) and very close to the wild type (W^1118^) [dCYFIP^85.1^, dFMR1^Δ113^=107.8 µm; dCYFIP^85.1^=69.8 µm (*P*=2.99×10^−9^) dFMR1^Δ113^=134.6 µm (*P*=2.39×10^−5^); *n*>30 for each case]. To get more insight into this interaction, we produced a single dose overexpression of dCYFIP in the *dFMR1* heterozygous background (*elav gal/+; UAS-dCYFIP/+; dFMR1^Δ113/+^*; Fig. S1B), which triggers a highly increased NMJ length, when compared with NMJs that are either heterozygous or overexpressing one dose of dCYFIP (Fig. S1B) (*P*<0.01 for both cases). This suggests a stronger impact of dCYFIP in the absence of dFMR1. We also tested the effect of an excess of dFMR1 in a *dCYFIP*-deficient background: single-dose overexpression of dFMR1 in *dCYFIP* heterozygous larvae results in NMJs similar to those observed in larvae overexpressing a single dose dFMR1 in a wild-type genetic background (*P*>0.05) (Fig. S1C), meaning that altering dCYFIP level does not reinforce the *dFMR1* GOF phenotype. In summary, dFMR1 and dCYFIP interact genetically at the fly larval NMJ, where they have an opposite and dosage-dependent role on NMJ length.

### Silencing of *Cyfip1* in cultured primary neurons

To study the impact of the loss of function of CYFIP1, we downregulated CYFIP1 protein and mRNA levels by RNA interference in mice, bypassing the lethality of *Cyfip1* homozygous mutant mice during embryogenesis (Pathania et al., 2014). We cloned two different shRNAs directed against the mRNA of mouse *Cyfip1* (Sh89 and Sh18) and a control shRNA (ShScr) in the pLL3.7 vector, expressing the green fluorescent protein (GFP). With these vectors, we generated three lentiviruses that we used to infect 6DIV mouse cortical neurons. At 21 days *in vitro* (DIV), we prepared RNA and proteins from these neurons and we tested the expression of *Cyfip1* by RT-qPCR and by western blot. The mRNA level of *Cyfip1* was reduced 70-80% by Sh89 and 45-50% by Sh18 (Fig. S2A). A similar reduction was also observed for its protein level (Fig. S2B,C). Previous studies in neurons obtained from *Cyfip1^+/−^* or from transgenic *Cyfip1-*overexpressing mice focused on dendritic morphology. It appears clear that overexpression of *Cyfip1* results in complex dendritic arborization of hippocampal neurons (Oguro-Ando et al., 2015), whereas *Cyfip1* heterozygous mice display a mild reduction of the arborization of the same neurons (Pathania et al., 2014). A similar phenotype was also described in rat neurons depleted for *Cyfip1* (Kawano et al., 2005). To validate the efficacy of our knockdown cellular model, we performed Sholl analysis of 21 DIV mouse cortical neurons transduced with lentiviruses carrying either Sh89 or ShScr. This resulted in a reduced arborization of infected neurons starting from 35-40 mm until 130 mm (Fig. S2D-F). The complexity of dendritic arborization is here profoundly reduced if compared with the reduced arborization of 14 DIV *Cyfip1*-depleted neurons as shown by other authors, where higher residual expression of *Cyfip1* is present (Pathania et al., 2014).

It was already known that the correct stoichiometric relationship between the different components of the WRC is critical for the stability of the complex. Indeed, it is reported that reduction of *Cyfip1* or fly *Hspc300* affects the level of the other components of the WRC (Kunda et al., 2003; Schenck et al., 2004; Abekhoukh and Bardoni, 2014; Gautier et al., 2011; Qurashi et al., 2007; De Rubeis et al., 2013). We confirmed these data by observing a reduced expression of the CYFIP2 protein (Fig. S3).

### Study of *in vivo* spine morphology

The tool we generated to reduce *Cyfip1* levels appears to be effective to highlight phenotypes that would probably not be observed if the residual expression of *Cyfip1* is still high. Starting from this consideration, we decided to use the lentivirus that expresses Sh89 (producing the highest level of reduction of *Cyfip1* mRNA) to knock down *Cyfip1* and to study the role of this gene *in vivo* in the presence and in the absence of its interactor FMRP. To this aim, we used the differentiation of granule cells (GCs) in the adult olfactory bulb (OB) as an *in vivo* model of neuronal maturation, which is a robust system for these analyses, as we have previously shown (Scotto-Lomassese et al., 2011; Daroles et al., 2015).

**Fig. 2.**
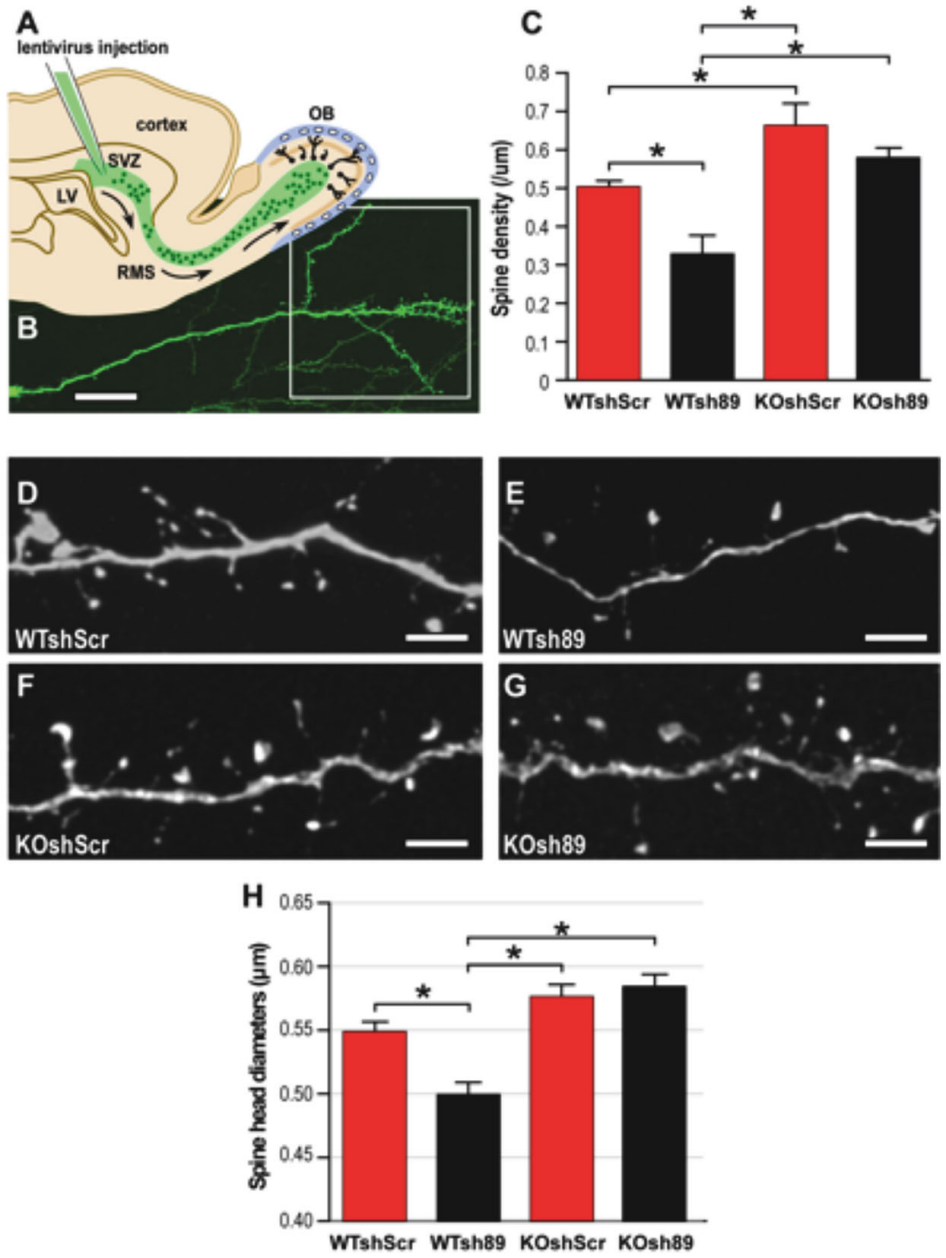
**Depletion of *Cyfip1* in neurons of the** **mouse** **olfactory bulb.** (A) Scheme of a sagittal section of the mouse forebrain. The subventricular zone (SVZ) of the lateral ventricle (LV) continuously produces new neurons, which migrate along the rostral migratory stream (RMS) and differentiate as interneurons in the olfactory bulb (OB). Subpopulations of young neurons can be labeled through stereotaxic injections of GFP-expressing viruses into the SVZ, which allow their morphological analysis. (B) GFP-labeled newly formed granule cell (GC) of the OB. GCs are anaxonic GABAergic interneurons with a long apical dendrite branching out into a dendritic arbor (outlined square). Scale bar: 100 µm. (C) Spine density in the dendritic arbors of new GCs in wild-type and *Fmr1* KO mice infected with ShScr or Sh89 (two-way ANOVA: genotype effect, *F*_1,37_=23.9, *P<0.0001; Sh effect, *F*_1,37_=10.23, *P=0.003; no genotype-Sh interaction; followed by LSD *post hoc* test, *n*=11,10,11,9). *Cyfip1* knockdown induces a downregulation of spine density in WT mice (difference between WT shScr and WT sh89, *P=0.004). In *Fmr1* KO mice, spine density of control neurons was increased compared with WT mice (difference between WT shScr and KO shScr, *P=0.009). *Cyfip1* knockdown has no effect on this increased density (no differences between KO shScr and KO sh89). (D-G) Representative images showing spines of the dendritic arbor of GFP-labeled new GCs in WT (D,E) or *Fmr1* KO mice (F,G) infected with viruses expressing a scrambled shRNA (ShScr; D,F) or an oligo directed against *Cyfip1* (Sh89; E,G). Scale bars: 5 µm. (H) Spine head diameters of ShScr- and Sh89-infected new neurons in WT or *Fmr1* KO mice. The absence of *Cyfip1* in new neurons induces a significant shift towards smaller diameters. The absence of *Cyfip1* in new *Fmr1*-mutated neurons does not influence spine head size (two-way ANOVA: genotype effect, *F*_1,37_=3,32, *P*=0.077; Sh effect, *F*_1,37_=1.52 *P*=0.225; genotype-Sh interaction, *F*_1,37_=7.92, *P=0.008, followed by LSD *post hoc* test, *n*=11,10,11,9).

We labeled and mutated a set of newly generated neurons in 3-month-old WT or *Fmr1-*knockout male mice by injection of viruses expressing Sh89 or a control (ShScr) in their area of production, the sub-ventricular zone (SVZ) (Fig. 2A,B). Mice were sacrificed 21 days after injection and the morphology of GFP-positive new neurons was subsequently studied, as previously described (Scotto-Lomassese et al., 2011; Daroles et al., 2015).

We analyzed spine density by counting protrusions in the dendritic arbor (Fig. 2B) of labeled GCs. In wild-type mice infected by the control ShScr virus, spine density of neurons was 0.51±0.02 spines/µm (Fig. 2C,D). This density was significantly decreased to 0.33±0.05 spines/µm in Sh89-infected new neurons (Fig. 2C,E). This suggests that *Cyfip1* plays a role in upregulating spine production, consistent with its role in actin remodeling. In *Fmr1*-null mice (Fig. 2C,F), the density of spines in control ShScr-infected neurons was significantly increased to 0.66±0.06 spines/µm compared with ShScr-infected neurons in normal mice (Fig. 2C,F), as we already described (Scotto-Lomassese et al., 2011), which suggests that FMRP plays a role in downregulating spine density. The number of dendritic spines of the knockout sh89-injected mice (KO sh89; Fig. 2G) is intermediate between that of WT mice injected with shScr (WT shScr; Fig. 2D) and that of KO shScr (Fig. 2F), but not statistically different from either (Fig. 2C), whereas the numbers of dendritic spines of WT sh89 and KO shScr (Fig. 2E,F) were significantly different from WT shScr (Fig. 2C,D). This means that in the absence of *Fmr1* (KO sh89) (Fig. 2C,G), *Cyfip1* knockdown did not trigger the decrease in spine density we observed in the presence of *Fmr1* (WT sh89) (Fig. 2C,E). Collectively, these data indicate an antagonistic effect of FMRP and CYFIP1 in the regulation of spine density. In addition, as for *Drosophila*, the incomplete rescue in the double depletion suggests that this phenotype is due to the antagonistic regulation of two (or more) pathways by FMRP and CYFIP1.

We then analyzed the spine morphology of *Cyfip1*-knockdown neurons in WT or *Fmr1-* knockout mice. GC spines display atypical morphologies with a long neck and variable head diameters. As previously described (Daroles et al., 2015), we did not follow the typical categorization of ‘mushroom’, ‘stubby’ and ‘thin’ spines, but we present the data here as spine head diameter (Fig. 2H). In wild-type mice, *Cyfip1* knockdown (WTsh89) triggered a significant shift towards smaller diameters in new neurons. Since *Fmr1-*null neurons (KO shScr) display spines with normal heads, we could not further support the antagonistic function of these two proteins in this context. In addition, *Cyfip1* knockdown did not have any effect on the spine head diameters of new neurons in *Fmr1*-knockout mice (Fig. 2H) suggesting that the residual expression of CYFIP1 does not allow to observe its effect in the absence of FMRP or, alternatively, a more complex interaction between these two proteins exists (see Discussion). We also tested the length of spines and did not observe any variation in any of the conditions we analyzed (Fig. S4).

### Molecular pathways regulated by the CYFIP1-FMRP complex

**Fig. 3.**
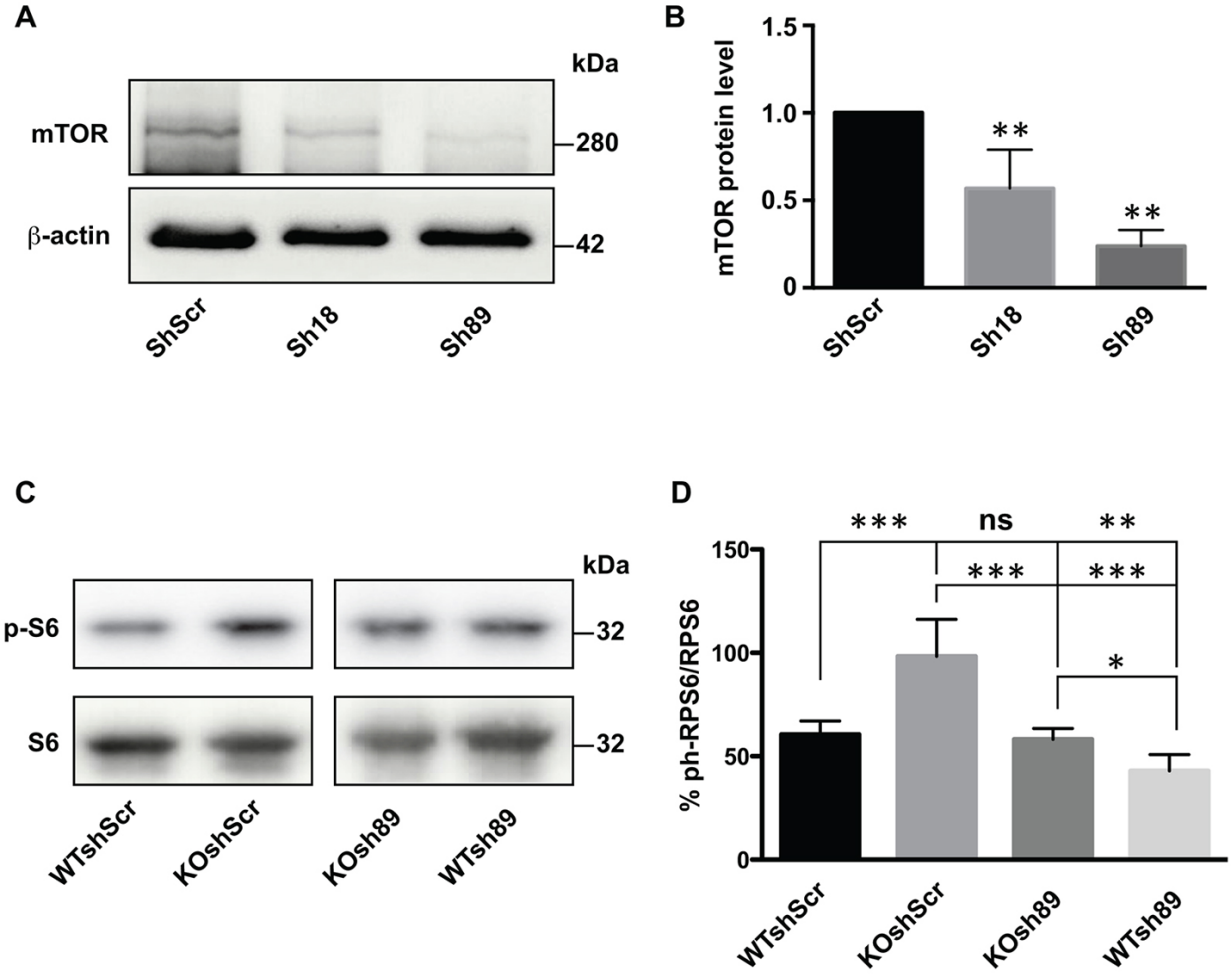
**Molecular analysis of the impact of *Cyfip1* depletion on the mTor pathway in the presence or in the absence of FMRP.** (A) Representative western blot analysis of cell cultures of cortical neurons transduced with ShScr, Sh18 or Sh89, respectively. The proteins detected are indicated on the left. The blot presented here was performed on the same membrane shown in Fig. S2B. (B) Densitometric analysis showing a significant reduction of mTor protein expression level in cell culture of cortical neurons transduced with ShScr, Sh18 or Sh89, respectively. Mean±s.e.m. of *n*=4 experiments is shown. Mann-Whitney test (**P<0.01). (C) Representative western blot analysis of cell cultures of cortical neurons obtained from normal or *Fmr1*-null mice and transduced with ShScr, or Sh89, respectively. The proteins detected are indicated on the left side of the blots. (D) Densitometric analysis showing pS6/S6 protein ratios of cell cultures of cortical neurons obtained from normal or *Fmr1*-null mice and transduced with ShScr or Sh89, respectively. Results are presented as the mean±s.e.m. of *n*=9 experiments; Tukey‘s multiple comparisons test (***P<0.001, **P<0.01, *P<0.05; ns, not significant).

**Fig. 4.**
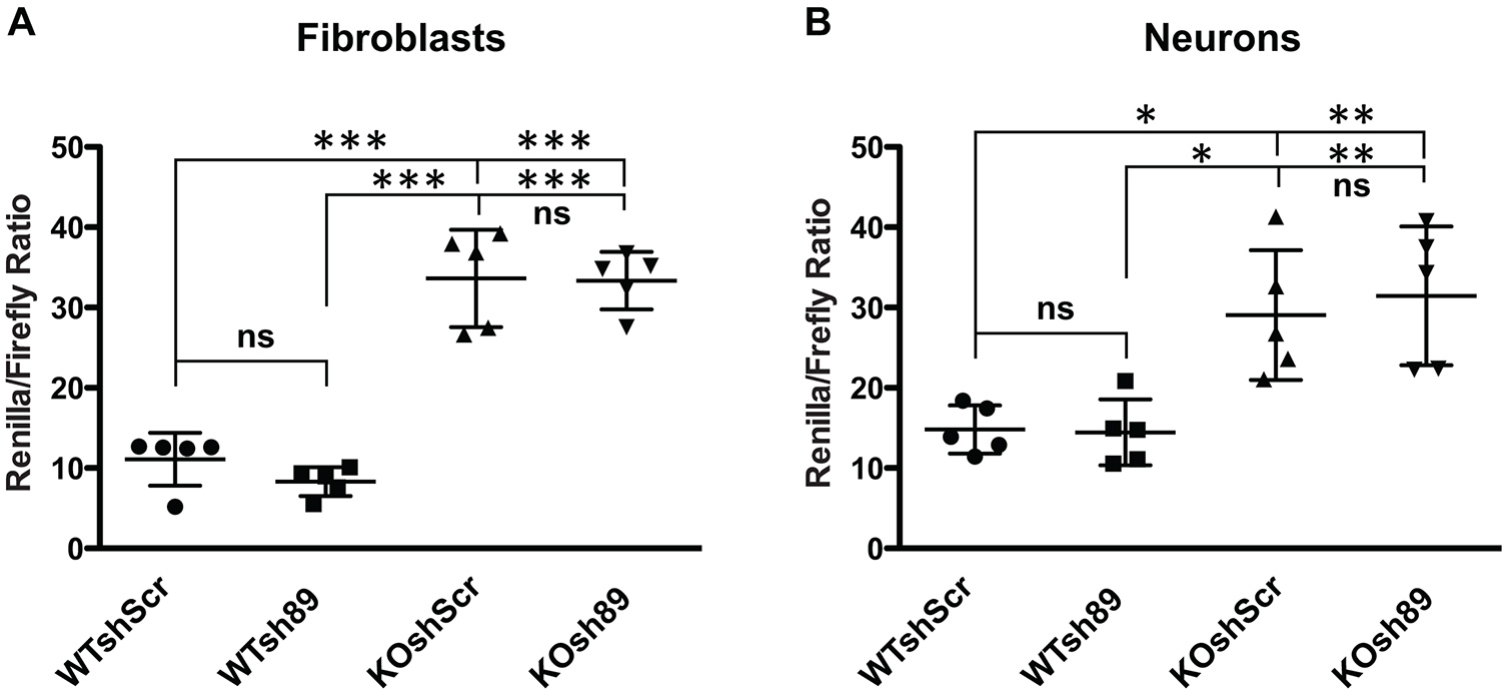
**Influence of FMRP and CYFIP1 on G-quadruplex-dependent translation.** (A) pRLTK plasmids expressing *Renilla* with or without a G-quadruplex RNA structure on its 5′UTR were transfected in mouse STEK cells (59 expressing *FMR1* ISO1 isoform and 87 that are *Fmr1*-KO) (Castets et al., 2005; Bechara et al., 2009; Maurin et al., 2015) transduced with ShScr or Sh89, respectively. These cells were co-transfected with a plasmid expressing firefly luciferase. The luciferase activities were measured and *Renilla* luciferase activity was normalized to firefly activity. Five experiments were carried out. Tukey's multiple comparisons test (***P<0.001). (B) The same experiment as described in A was performed in mouse WT and *Fmr1*-KO cultured cortical neurons. Five experiments were carried out. Tukey's multiple comparisons test (**P<0.01; *P<0.05). Individual data points along with mean±s.e.m. are shown.

The results we obtained using *Drosophila* as an animal model provided evidence for a strong genetic interaction between CYFIP and dFMR1. It was shown that an increased level of *Cyfip1* correlates with an enhanced expression of mTor (Oguro-Ando et al., 2015) at the mRNA and protein levels, suggesting a role of CYFIP1 in mRNA stability or, indirectly, transcriptional regulation. With this premise, we tested the level of mTor protein in extracts of cultured *Cyfip1*-knockdown primary cortical neurons and we observed a 40-60% reduction (Fig. 3A,B). Conversely, it was reported that the mTor pathway is enhanced in *Fmr1*-knockout neurons (Sharma et al., 2010; Sawicka et al., 2016). This alteration is not due to an altered expression of mTor (Sharma et al., 2010), but rather to the overactivation of PI3K, because of the overexpression of the catalytic PI3K subunit p110β (Gross et al., 2010; Gross and Bassell, 2012). However, the expression level of this latter protein is not influenced by CYFIP1, as we show in Fig. S5. Since this represents a possible example of antagonistic regulation of different pathways converging to mTor signaling, we studied the impact of the reduced expression of *Cyfip1* on downstream mTor signaling in the presence and in the absence of FMRP. To this purpose, we monitored the phosphorylation level of the S6 ribosomal protein (pS6/Rps6), a well-known target of mTor signaling (Magnuson et al., 2012) in cultured cortical neurons obtained from WT and from *Fmr1*-null mice transduced with lentivirus expressing Sh89 or a control shRNA. We observed an increased level of pS6 in *Fmr1-*null neurons (KO shScr; Fig. 3C,D) and in line with the antagonistic action of the two proteins, we observed a reduced phosphorylation level in neurons obtained from control mice where *Cyfip1* was knocked down (WT sh89; Fig. 3C,D). Interestingly, a normal level of phosphorylation of S6 was restored when *Cyfip1* was knocked down in *Fmr1*-null cells (KO sh89; Fig. 3C,D). This finding shows a positive role for CYFIP1 in pS6-mediated translational regulation, which is an opposite function with respect to FMRP (Sharma et al., 2010; Maurin et al., 2014). In this case, the two proteins may act independently on the same pathway. In addition, we compared the impact of FMRP and CYFIP1 on G-quadruplex-dependent translation. G-quadruplex mRNA is a structure bound by FMRP harbored by some of its critical targets (*MAP1B*, *PP2AC*, *FMR1*, *SHANKL*, *SEMA3F*) (Brown et al., 2001; Castets et al., 2005; Schaeffer et al., 2001; Zhang et al., 2014; Menon and Mihailescu, 2007). Moreover, the G-quadruplex/FMRP complex mediates the repression activity of FMRP in translational regulation (Schaeffer et al., 2001; Brown et al., 2001; Castets et al., 2005; Melko and Bardoni, 2010; Suhl et al., 2015). A vector expressing luciferase under the control of a G-quadruplex structure in its 5′UTR (Schaeffer et al., 2001) was used to transfect fibroblast cell lines (Stek cell lines) (Castets et al., 2005; Bechara et al., 2009; Maurin et al., 2015) either expressing or not expressing FMRP and where *Cyfip1* was either depleted or not. As expected (and as shown in Schaeffer et al., 2001), in *Fmr1*-null cells, the expression level of luciferase is increased because of the absence of the repression activity of FMRP (Fig. 4A). However the knockdown of *Cyfip1* does not change luciferase activity levels (Fig. 4A). We repeated the same experiment in 14 DIV primary cultured cortical neurons and we also confirmed the repressor activity of FMRP in the presence of a G-quadruplex structure, but we did not observe any impact of CYFIP1 on luciferase activity in either the presence or absence of FMRP (Fig. 4B).

We can thus conclude that the co-repressor activity of CYFIP1-FMRP does not affect G-quadruplex-containing mRNAs, which represent a subset of FMRP targets (Brown et al., 2001; Maurin et al., 2014; Suhl et al., 2015).

### Expression level of mRNA of WRC members

**Fig. 5.**
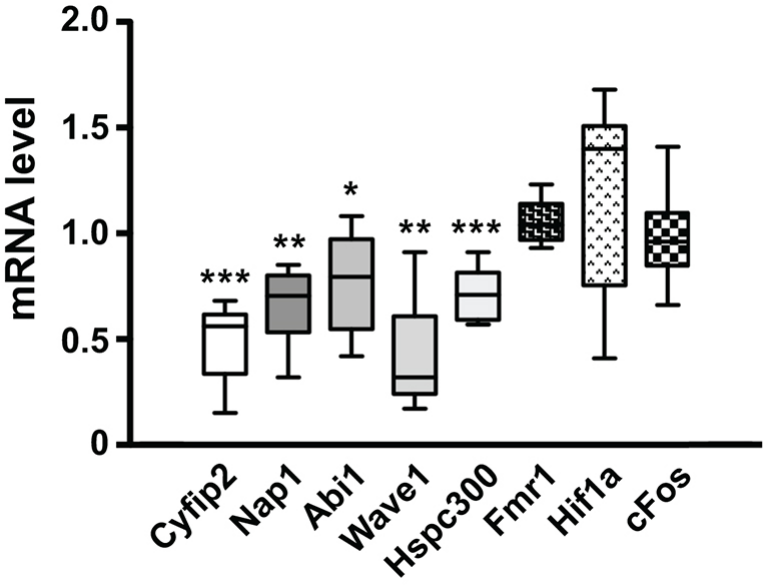
**mRNA levels of WRC members in *Cyfip1*-depleted neurons.** Total mRNA was prepared from cultured 21 DIV cortical neurons transduced at 6 DIV with ShScr or Sh89. Levels of mRNAs of *Cyfip2*, *Nap1*, *Abi1*, *Wave1*, *Hspc300* and three control mRNAs: *Fmr1*, *cFos* and *Hif1a* were measured by RT-qPCR. Eight different experiments were carried out. Results are presented as ratios of the values of Sh89-transfected neurons over ShScr-transfected neurons for each mRNA and are presented as the mean±s.e.m.; Mann-Whitney test (***P<0.001; **P<0.01; *P<0.05). The level of *Cyfip1* in these experiments is shown in Fig. S2.

**Fig. 6.**
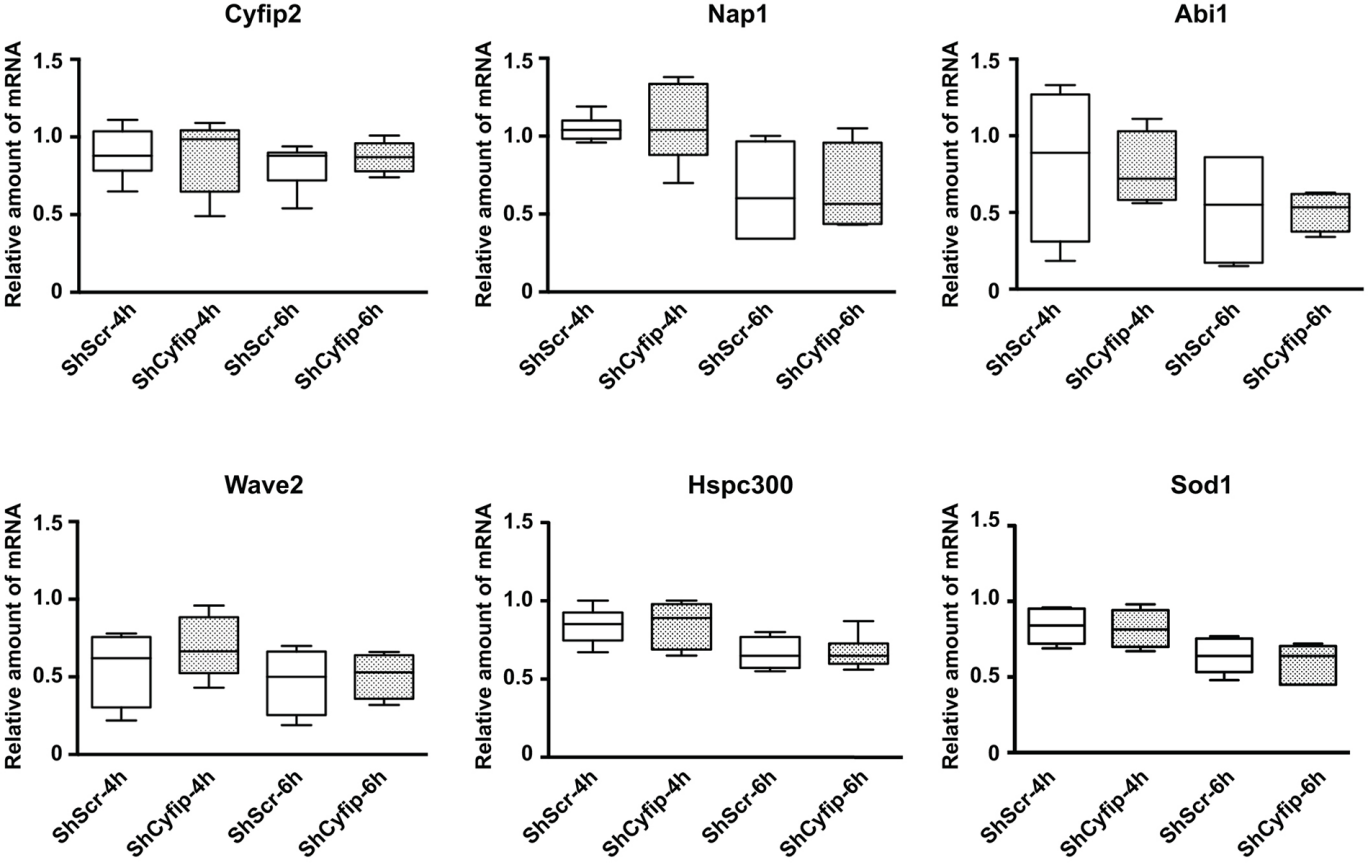
**mRNA stability of WRC members in mouse *Cyfip1-*depleted cortical neurons.** 21 DIV primary cultured cortical neurons transduced at 6 DIV with lentiviruses expressing the following shRNAs: ShScr, Sh89 or Sh18 were incubated with 5 mM actinomycin D. Total RNA was extracted at T0 and at 4 h and 6 h after the treatment. *Cyfip2*, *Nap1*, *Abi1*, *Wave1*, *Hspc300* and *Nipa2* (control) mRNAs were quantified by RT-qPCR, comparing each time with the corresponding T0. Six experiments were carried out: four using Sh89 and two using Sh18. Results are presented as the mean±s.e.m.; Mann-Whitney test. No significant statistical differences were observed.

As mentioned above, levels of CYFIP1/dCYFIP proteins influence the levels of the other components of the WRC (Schenck et al., 2004; Abekhoukh and Bardoni, 2014; Gautier et al., 2011). We could thus hypothesize that CYFIP1 (or dCYFIP in the fly) is not stable when the precise stoichiometry of each WRC component is not respected. However, the overexpression of *Cyfip1* generated a phenotype in *Drosophila* (Fig. 1B) as well as in mice neurons (Oguro-Ando et al., 2015; Pathania et al., 2014) in the opposite manner to that observed upon its depletion (Pathania et al., 2014; this study). We decided to measure mRNA levels of WRC members by real-time PCR in *Cyfip1*-knockdown neurons. We were surprised to find that the levels of these mRNAs were all reduced, while the level of *Fmr1* mRNA was unchanged (Fig. 5). We then tested the expression levels of these same mRNAs in neurons where the *Cyfip1* level was reduced by Sh18. We confirmed the reduced mRNA expression level of all the WRC members in *Cyfip1* mutants (Fig. S6). To explain the reduced levels of the WRC mRNAs in *Cyfip1*-depleted cells, we asked whether the half-life of these mRNAs is modified when *Cyfip1* expression is knocked down. We treated 21 DIV neurons with 5 mM actinomycin D and after 4 and 6 h of treatment, we evaluated the reduction in the mRNA levels of *Cyfip2*, *Nap1*, *Abi1*, *Wave2*, *Hspc300* and *Sod1* as a control (Fig. 6), in the presence of *Cyfip1* (neurons transduced with the lentivirus expressing ShScr) or after its knockdown (neurons transduced with the lentivirus expressing Sh89 or Sh18). No differences were observed between these conditions, suggesting that the different mRNAs have the same half-life in the presence or absence of *Cyfip1* and that the co-regulation of these mRNAs is not carried out at the post-transcriptional level.

### WRC expression in patients carrying the BP1-BP2 deletion

The human *CYFIP1* gene is located on 15q11.2, a chromosomal region in the recurrent BP1-BP2 deletion (Abekhoukh and Bardoni, 2014). As for *Cyfip1*-depleted neurons, we analyzed the mRNA levels of the WRC members in total RNAs from four lymphoblastoid cell lines obtained from North American patients carrying the BP1-BP2 deletion (see supplementary Materials and Methods for genotype and phenotype description) and compared it with RNA from four lymphoblastoid cell lines obtained from normal subjects. We confirmed a significant reduction of all WRC member mRNAs (Fig. S7A) with the exception of WAVE2, whose very small reduction was not statistically significant (*P*=0.3). Since lymphoblastoid cells are not primary cell lines and the expression of genes can be modified by immortalization, we analyzed the expression level of WRC mRNAs in the blood of five patients belonging to four different families (from different geographic origins, Europe and South America, respectively) all carrying the BP1-BP2 deletion on chromosome 15q11.2 (see supplementary Materials and Methods) encompassing the gene encoding CYFIP1. In all patients analyzed, a significant reduction of the *CYFIP1* mRNA level was detectable, accompanied by a decreased level of *CYFIP2*, *NAP1*, *ABI1*, *WAVE2* (the only WAVE member expressed in blood cells) and *HSPC300* mRNAs compared with the four controls (Fig. S7B). We also analyzed a rare case of 4× BP1-BP2 (instead of 2× as in controls; Fig. S7C), observing that the mRNA levels of the WRC members tended to be increased in this patient when compared with the same four controls we used in the analysis of patients carrying the deletion (Fig. S7B). Although not conclusive because we used only a single patient (see supplementary Materials and Methods), all values (with the only exception of *ABI1* in only one control) were lower for controls compared with the patient (Fig. S7C) and the Crawford and Howell test (a modified unilateral *t*-test) (Crawford and Howell, 1998) supported this trend (Fig. S7C). Collectively, these results suggest that there is a direct correlation between the mRNA levels of *CYFIP1* and those of the WRC members.

## DISCUSSION

Despite the multiple interactions of CYFIP1 and FMRP, the CYFIP1-FMRP complex has only been described in co-regulation of translational initiation (Abekhoukh and Bardoni, 2014). In this context, we used two animal models (mice and flies) to better understand the role of CYFIP1.

### Altered expression levels of the mRNAs of WRC components

If post-translational regulation determines the stoichiometry of the WRC members, as previously suggested by several authors (Kunda et al., 2003; Schenck et al., 2004), no phenotype should be observed after *Cyfip1* overexpression. In patient cells and mouse neurons (Oguro-Ando et al., 2014; Pathania et al., 2014), as well as in the fly NMJs (this study), it was observed that overexpression of *Cyfip1/dCYFIP* is possible and the phenotypes observed are in general the opposite of those observed after the depletion of the same gene. For this reason and considering that new mechanisms of co-regulation of gene members of the same network have been proposed (Tay et al., 2014; Karreth et al., 2014; see below), we predicted a (co-)regulation of WAVE members that is not only dependent on proteasomal degradation, as previously proposed (Schenck et al., 2004). With this premise, we were not surprised to observe that reduced expression of *Cyfip1* mRNA is correlated to the reduced mRNA levels of all WRC members. This effect is more obvious when the *Cyfip1* mRNA level is reduced by more than 50% compared with control cells, with a single exception in immortalized cell lines (Fig. 5; Fig. S7). By contrast, amplification of the BP1-BP2 region resulted in a tendency towards increased expression of the WRC members (Fig. S7). This result suggests a co-regulation of WRC members as occurs for the components of the mitochondrial proteins involved in energy production, which form a multimeric OXPHOS complex and are co-expressed and co-regulated at the transcriptional level (van Waveren and Moraes, 2008). Indeed, the presence of common transcriptional regulatory elements in the promoters of all WRC members supports this hypothesis. Possibly, this co-regulation takes place at the transcriptional level since no difference in the half-life of the mRNAs coding for the WRC components is observed in *Cyfip1*-depleted neurons. Since the WRC mRNAs are common targets of a subset of microRNAs (www.microrna.org/microrna/home.do), we can hypothesize that these genes are part of a network of regulation, as proposed for other mRNAs co-regulated by the same microRNAs (Tay et al., 2014). The same mechanism of gene network regulation might be extended to a co-regulation by common factors involved in their stabilization, but also in transcription due to their limiting amount, as recently proposed (Karreth et al., 2014). In any case, we cannot support a proteasomal pathway regulation of WRC members as the unique mechanism to explain their stoichiometric regulation. Thus, our data highlight the importance of a tight regulation of the components of this complex that is critical for cell function.

### Neuronal impact of the depletion of *Cyfip1* in mouse

In mice, the reduced number of spines that we observed in knocked-down *Cyfip1* adult GC neurons is consistent with the observation that overexpression of *Cyfip1* produces an increased number of dendritic spines (Oguro-Ando et al., 2014) and with previously described phenotypes after depletion of other members of the WRC complex. Indeed, the number of dendritic spines is reduced in mice null for *Wave1* (Hazai et al., 2013) and *Abi2* (Grove et al., 2004)*.* For this reason it is surprising that other studies performed in *Cyfip1^+/−^* mice reported an unchanged density of spines in cortical and hippocampal cultured neurons (Pathania et al., 2014; De Rubeis et al., 2013). It is thus possible that only a strong reduction of the Cyfip1 expression level (not reached in those studies) would allow the phenotypes or their severity to be uncovered (consistent with the dosage-dependent phenotype we observed in *Drosophila*) or, alternatively, depletion of *Cyfip1* has a different impact in different brain regions as also shown for *Cyfip2* (Han et al., 2014).

Interestingly, the smaller spine heads observed when we depleted *Cyfip1* (Fig. 2) might be interpreted as a prevalence of immature spines (Dailey and Smith, 1996; Yuste and Bonhoeffer, 2004). However, since these spines have a normal length they cannot be considered as immature filopodia, raising the question of whether the small heads can be due to a prevalence of an altered structure caused by: (i) altered actin dynamics (consistent with reduced levels of WRC members) and/or (ii) altered translational regulation (consistent with reduced phosphorylation S6). Other studies have proposed that the reduced expression of *Cyfip1* (using heterozygous *Cyfip1^+/−^* mice) resulted in immature spines at 14 DIV in cultured cortical (De Rubeis et al., 2013) and hippocampal (Pathania et al., 2014) neurons, while the overexpression of *Cyfip1* resulted in an increased number of abnormal spines (Oguro-Ando et al., 2015) or, surprisingly, immature spines (Pathania et al., 2014). Furthermore, the concept of immature spines also depends on which theory of synaptogenesis is considered: spinogenesis-dependent (independent of axon-dendrite contact) (Miller, 1988; Sotelo, 1991; Yuste and Bonhoeffer, 2004), or spinogenesis starting from a filopodium (Dailey and Smith, 1996; Yuste and Bonhoeffer, 2004). Taking this issue into consideration, some spine morphological aspects (e.g. number, size and length) can be more objective parameters than the analysis of putative immaturity. Thus, differences in these conditions may explain the different results obtained by various laboratories in the description of *Cyfip1*-depleted neurons. All these considerations support the use of an already validated *ex vivo* system (Scotto-Lomassese et al., 2011; Daroles et al., 2015), as we used here.

### Relationship between CYFIP1 and FMRP

We used two animal models to get further insights into the functional significance of the FMRP-CYFIP1 interaction. First, in the fly we confirmed that CYFIP and dFMR1 affect synapse growth in opposite ways. Interestingly, the overexpression of CYFIP promotes excessive synapse growth (Fig. 1; Fig. S1). Since we have shown that CYFIP is stable only if part of the WRC complex (Schenck et al., 2004), this result suggests that the levels of WRC members should be tightly co-regulated, as we propose here in mouse and human. In addition, the double mutant data reveal two important aspects of CYFIP-dFMR1 interaction: (i) the finding that CYFIP and dFMR1 mutants can rescue each other's defects suggests that they control a common pathway and/or two antagonizing pathways; (ii) the combination of the two mutants results in an NMJ length significantly different from both single mutants, suggesting that, for synapse growth, one phenotype cannot dominate the other one. Similarly, an excess of CYFIP reinforces the dFMR1 heterozygous phenotype, suggesting that dFMR1 may control CYFIP activity. However, after compromising the CYFIP level, no reinforcement of the dFMR1 gain-of-function phenotype is observed, suggesting that CYFIP is only partially controlled by dFMR1. We can conclude that, at the molecular level, a two-sided link might exist between the two molecules underpinning their antagonistic action.

In the mouse neuronal model, the phenotype (number of dendritic spines) generated by *Cyfip1* depletion in GCs is opposite to that observed when *Fmr1* is depleted. This hallmark is rescued in mouse GCs double-depleted for *Fmr1* and *Cyfip1* (Fig. 2C), similar to NMJ length in the fly double knockout (Fig. 1D). This conclusion is consistent with the observation that the mTor pathway is antagonistically regulated by CYFIP1 and FMRP (Fig. 3). The fact that the number of dendritic spines in the double knockout is intermediate between wild-type and *Fmr1*-knockout mice, not appearing significantly different from both values, supports the idea that the two proteins act independently and antagonistically on one or more pathways to define this phenotype. This hypothesis is also supported by the observation that a complete rescue is not possible. In addition, in mouse, the depletion of *Cyfip1* is not as complete as in a knockout model. By analogy and homology with the fly and considering the molecular results we obtained, we would like to exclude the possibility that the *Fmr1*-knockout phenotype can dominate the *Cyfip1*-knockdown phenotype. On the other hand, the absence of FMRP does not modify the wild-type ‘size of spine heads’ phenotype, and the depletion of *Cyfip1* does not impact this phenotype, leading to a couple of possibilities: (i) this phenotype is probably revealed only by the presence of the double knockout (*Cyfip1* is knocked down in this experiment), as in the fly where a dosage-dependent phenotype is observed; (ii) the FMRP phenotype dominates the CYFIP1 phenotype in this context and, at the molecular level, the function of CYFIP1 depends on the presence of FMRP. This effect could be due to the fact that the absence of FMRP is likely compensated by FXR1P and/or FXR2P, as reported for other phenotypes (Zhang et al., 2008). CYFIP1 does not interact with either FXR1P or FXR2P and its absence cannot affect their function as it does for FMRP.

The CYFIP1-FMRP complex is involved in the definition of different pathways impacting neuronal morphology. These pathways appear to be modulated in different ways by this protein complex, probably depending on the presence of other partners or specific inputs. For instance, in the double homozygous mutant, the neuromuscular junctions are still significantly longer than in the wild type [dFMR1^Δ113^, CYFIP^85.1^ (107.8) versus dFMR1^W1118^ (93.5); *P*=0.006] leading to the final conclusion (only possible in this experiment) that additional molecules counteract FMRP. It is interesting to notice that in mouse, while we are describing CYFIP1 as an antagonist of FMRP, CYFIP2 has been described both as a co-effector (e.g. depletion of CYFIP2 worsens some phenotypes of *Fmr1*-knockout mouse) (Han et al., 2015) and a downstream target of FMRP. Indeed, in the mouse brain, *Cyfip2* mRNA (but not *Cyfip1* mRNA) was reported to be a target of FMRP that represses its translation (Darnell et al., 2011). Overall, the role of CYFIP1 cannot only be restricted to the unique function of acting as a co-regulator of the initiation of translation together with FMRP (Napoli et al., 2008). Indeed, FMRP is not only involved in translational repression, it is also a translational enhancer (Bechara et al., 2009; Gross et al., 2011; Kwan et al., 2012; Tabet et al., 2016) and a translation-independent function was also described for FMRP (Myrick et al., 2015). In addition, this protein is involved in RNA transport along neurites and between nucleus and cytoplasm (Maurin et al., 2014). The role of CYFIP1/2 in FMRP functions other than repression of translational initiation was never explored (Maurin et al., 2014), while it is known that CYFIP1 interacts with many proteins (Rac, WRC members and membrane components) that are not known partners of FMRP (Abekhoukh and Bardoni, 2014).

### Functional significance of the FMRP-CYFIP interaction

The CYFIP1-FMRP interaction, initially identified by two-hybrid screening in yeast (Bardoni et al., 1999; Schenck et al., 2001), was confirmed by GST pull-down and immunoprecipitation both in mouse and fly cell lines and tissues (Schenck et al., 2001, 2003; De Rubeis et al., 2013). CYFIP1 binds both FMRP and eIF4E, suggesting a critical role as co-repressor (together with FMRP) of translational initiation. It was reported that BDNF regulates translation by causing release of CYFIP1 from eIF4E via the action of MAP kinase interacting serine/threonine kinase 1 (MNK1) (Napoli et al., 2008; Panja et al., 2014). In particular, in *Mnk1*-null neurons, altered expression of a subset of proteins was observed, including 26 proteins whose encoding mRNAs are bound by FMRP in translating ribosomes (Genheden et al., 2016; Darnell et al., 2011). Here, we show that this latter regulation is not valid for G-quadruplex-containing mRNAs. RNA G-quadruplex-forming structures are involved in several steps of mRNA metabolism (Melko and Bardoni, 2010; Subramanian et al., 2011). In particular, the G-quadruplex is known to have per se a role in translational repression. This function is reinforced by the presence of FMRP (Kumari et al., 2007; Melko and Bardoni, 2010). This probably happens because the FMRP-G-quadruplex complex tightly blocks the advance of polyribosomes (Melko and Bardoni, 2010; for review). The level of luciferase encoded by an mRNA harboring a G-quadruplex in its 5′UTR in the presence of FMRP is not modified upon CYFIP1 depletion (Fig. 4). Conversely, in *Fmr1-*null cells (both neurons and fibroblasts), the absence of FMRP removes the translational repression that is, again, not dependent on the presence of CYFIP1. In conclusion, G-quadruplex-containing FMRP target mRNAs are not repressed via interaction with the CYFIP1-eIF4E subcomplex, as proposed for other targets of FMRP (Napoli et al., 2008). Moreover, our data leading to the conclusion that CYFIP1/dCYFIP and FMRP/dFMR1 can also act antagonistically and independently, are supported at the molecular level by results showing an antagonistic regulation of the mTor signaling via different pathways involving FMRP and CYFIP1. This result raises the question concerning the functional role of the interaction of these two proteins if they also act independently. Since their action is antagonistic, we predict that a tight coordination is needed. In this context, we support the hypothesis that CYFIP1/dCYFIP might be an intermediate messenger protein linking FMRP/dFMR1 to actin remodeling and/or other signaling, likely coordinating these processes. Indeed, GTP-Rac binds CYFIP/CYFIP1, modifying its structural conformation (Kobayashi et al., 1998; De Rubeis et al., 2013) and allowing it (together with NCKAP1 and ABI1) to leave the WRC and interact with other factors. At the same time, the rest of the WRC complex resulting from the scission (e.g. WAVE and HSPC300) is actively involved in actin polymerization, together with Arp2/3 (Cory and Ridley, 2002). Our model does not exclude the existence of a FMRP-CYFIP1-eIF4E subcomplex negatively regulating the translation of a subset of mRNAs (Napoli et al., 2008; De Rubeis et al., 2013). However, the precise mechanism of this regulation should be probably better dissected, as also suggested previously by other authors (Iacoangeli et al., 2008).

### Relationship between CYFIP1 and CYFIP2

Owing to their high level of homology, CYFIP1 and CYFIP2 have been often considered as having the same function (Abekhoukh and Bardoni, 2014). In *Fmr1*-knockout mice the *Cyfip2^+/−^* neuronal phenotype worsened (Han et al., 2015), while our data shows an antagonistic role of CYFIP1 and FMRP. Taken together, these results underline different functions of CYFIP1 and CYFIP2. Some findings from other laboratories are in line with our results. Indeed, (i) while both CYFIP1 and CYFIP2 interact with FMRP, only CYFIP2 interacts with FXR1P and FXR2P, the two paralogs and interactors of FMRP (Schenck et al., 2001); (ii) the expression of the two proteins appears differently modulated in different brain regions during post-natal brain development, while the three FXR proteins have the same expression pattern during development (Bonaccorso et al., 2015); (iii) depletion of CYFIP1 in epithelial cancer cells interferes with the morphology of the cells and induces invasion (Silva et al., 2009). This phenotype is also present when WAVE1 or NAP1 are knocked down. Conversely, knockdown of *CYFIP2* induces a dramatic reduction of proliferation while knockdown of *FMR1* does not have an impact on these cells (Silva et al., 2009). These data indicate that CYFIP1 and CYFIP2 have a different role in their interaction with FMRP and that dCYFIP looks functionally more similar to CYFIP1, even if it shares the same level of homology with both mammalian homologs.

### Conclusions

The initial hypothesis of an antagonist action of dCYFIP and dFMR1 is still valid in fly and mouse; however, our new analyses indicate a more complex scenario with a two-sided link between the CYFIP1/FMRP-containing pathways. In particular, it seems important to underline that according to our results, CYFIP1 and FMRP can also act independently and not only as translational co-repressors on a subset of genes. These conclusions are consistent with the fact that FMRP is, indeed, not only involved in translation regulation (Maurin et al., 2014) and CYFIP1 is a component of complexes that do not contain FMRP (Abekhoukh and Bardoni, 2014; Chen et al., 2014). Last but not least, CYFIP1 does not behave as a repressor of G-quadruplex-dependent translation.

We also show here a direct correlation between the mRNA levels of *CYFIP1* and those of the WRC members. This can link WRC and FMRP-containing complexes since altering the level/activity of CYFIP1 results in an altered antagonistic action on FMRP downstream pathways, possibly contributing to neuronal pathologies. Overall, this study suggests that new models and supplementary analyses are needed to decipher the role of CYFIP1 in the pathophysiology of neurodevelopment and, in particular, in the aetiology of neurodevelopmental disorders.

## MATERIALS AND METHODS

### Fly lines

Flies were raised at 25°C on standard food. *W^1118^* flies were used as controls. The *elav*-*Gal4 (C155)* line was provided by the Bloomington Stock Center (Bloomington, Indiana, USA). The *dCYFIP^85.1^* (Schenck et al., 2003) and *dFMR1*(*dFMR1^Δ113^*) (Zhang et al., 2004) lines were used as null alleles. The double mutant strain (*dFMR1^Δ113^, dCYFIP^85.1^ rec*) was obtained upon recombination. The *UAS-dFMR1* (generous gift from Y. Q. Zhang, Institute of Genetics and Developmental Biology, Chinese Academy of Sciences, Beijing, China) and the *UAS-dCYFIP* (Schenck et al., 2003) lines were used for the overexpression studies.

### Fly neuromuscolar junctions

Neuromuscular junctions (NMJs) were stained with the mouse anti-Discs-large (Dlg, DSHB, #4F3) primary antibody. Quantification of NMJs was performed essentially as previously described (Schenck et al., 2003). At least two rounds of independent assays were carried out per genotype; for each round, at least 10 late L3 larvae of normal body size were dissected and 30 NMJs were analyzed. In all cases, type-Ib NMJs on muscle 4 of abdominal segments A2-A4 were scored. Pictures were taken with a 40× objective on a Zeiss Axiophot 2 microscope and imported using an in-house developed TCS/time software that quantifies synaptic length by automatic measurement of synaptic terminals. Statistical significance was calculated using ANOVA and the Newman-Keuls method for *post hoc* pairwise analyses.

### Mouse and human samples

Mice were obtained from R. Willemsen and Ben Oostra, Erasmus University, Rotterdam, the Netherlands (Mientjes et al., 2006). Animal care was conducted in accordance with standard ethical guidelines (National Institutes of Health publication no. 85-23, revised 1985 and European Committee Guidelines on the Care and Use of Laboratory Animals 86/609/EEC). The experiments were approved by the local ethics committee (Comité d'Ethique en Expérimentation Animale Charles Darwin C2EA-05).

All human patient samples were obtained according to the guidelines of the ethic rules of medical centers involved in the study (CIBER of Rare Diseases, Barcelona, Spain; Instituto de Pesquisa Pelé Pequeno Principe, Curitiba, Brazil; Institute of Rare Diseases; Institute of Medical Genetics; The Chaim Sheba Medical Center, Tel Hashomer, Israel; Hospital for Sick Children, Toronto, Ontario, Canada) and written informed consent was obtained from all participants. Full details of patients from whom whole blood samples and lymphoblastoid cell lines were obtained and methods for genomic analysis are supplied in supplementary Materials and Methods.

### Cell culture

Cortical neurons were prepared from C57/BL6 wild-type mice at E15.5. Dissection was performed in cold Hank's balanced salt solution (HBSS; Invitrogen). Brains were quickly extracted from the skull and washed twice in cold HBSS. Striatum and meninges were removed and cortex dissociated in 500 µl of DMEM (DMEM 4.5 g/l, high glucose with L-glutamine; PAA Laboratories) and 10% fetal bovine serum (FBS). After total dissociation, medium was added to make 10 ml and samples were filtered with a cell strainer (70 µm). Cells were then centrifuged for 5 min at 1200 rpm and diluted in the neuron culture medium (Neurobasal medium, Gibco) supplemented with B-27 (Gibco) and antibiotics (5000 IU/ml penicillin and 5 mg/ml penicillin-streptomycin; PAA Laboratories) at a concentration of 10^6^ cells/ml. Neurons were seeded in sterile culture dishes of 60 mm diameter at a density of 10^6^ cells per dish for molecular analysis or in 24-well plates containing glass slides (14 mm in diameter) at a density of 50,000 cells per well for microscopic studies. Dishes and slides were previously coated with 0.04 mg/ml ornithine (Sigma Aldrich).

### Cell fixation

Neurons were infected at 6 DIV and then fixed at 21 DIV. After washing twice at room temperature with cold PBS, neurons were fixed for 1 h with binding buffer (3.7% formaldehyde and 5% sucrose in PBS), rinsed twice with PBS and then treated for 10 min with 50 mM NH_4_Cl at room temperature. Slides were rinsed twice with PBS and then mounted as previously described (Melko et al., 2013).

### Lentivirus generation

We designed oligonucleotides targeting *Cyfip1* (Sh89 and Sh18) or control sequence (scrambled or Scr):

Sh89 F: 5′-TGGCAATTGGACGGTTTGAATTCAAGAGATTCAAACCGTCCAATTGCCTTTTTTC-3′; Sh89 R: 5′-CTCGAGAAAAAAGGCAATTGGACGGTTTGAATCTCTTGAATTCAAACCGTCCAATTGCCA-3′; Sh8 F: 5′-TCGCTGCTCTATCAGCCAAATTCAAGAGATTTGGCTGATAGAGCAGCGTTTTTTC-3′; Sh18 R: 5′-CTCGAGAAAAAACGCTGCTCTATCAGCCAAATCTCTTGAATTTGGCTGATAGAGCAGCGA-3′; ShScr F: 5′-TTCGTCATAGCGTGCATAGGTTCAAGAGACCTATGCACGCTATGACGATTTTTTC-3′; ShScr R: 5′-CTCGAGAAAAAATCGTCATAGCGTGCATAGGTCTCTTGAACCTATGCACGCTATGAGAA-3′.

The oligonucleotides coding the different shRNAs were cloned in the pLL3.7 vector (Addgene) in the *Hpa*I and *Xho*I sites and correct insertion were verified by sequencing using the following primers: F: 5′-CCGGCAGCAGGCCGCGGGAAG-3′; R: 5′-ACTATTAATAACTAATGCATG-3′.

pLL3.7 is a third-generation lentiviral vector that expresses shRNA under the control of the mouse U6 promoter. A CMV-EGFP reporter cassette is included in the vector to monitor expression. Plasmid DNA was purified with a Qiagen kit (DNA Maxi, Midi, Mini Kits and Endofree-Maxi kit). Lentivirus vector particles were produced as previously described (Khalfallah et al., 2009). To titrate the virus, neurons were cultured at a density of 50,000 cells per well in a 24-well plate. Infection was carried out in a minimum volume of neuronal media at 6 DIV using serial dilutions of the virus. The virus concentration is chosen so that the infection of neurons is maximal for biochemical studies (ideally 100%) and lowest for studies of neuronal morphology (about 5%).

### RT-qPCR

mRNA expression levels were measured by RT-qPCR. The RNA was extracted using the RNeasy kit (Qiagen) from cultured neurons, lymphoblastoid cells, fresh blood or using the BioMaxi Blood RNA purification kit (Biomatrica) from the blood of patients collected in RNAgard blood tube (Biomatrica). Reverse transcription was performed on 1 µg RNA by SuperScriptIII (Invitrogen) following the manufacturer's instructions. The quantification of the level of the different mRNAs was carried out by quantitative PCR using specific primers. qPCR was carried out using the LightCycler 480 Real-time PCR system (Roche) with SYBR Green qPCR core kit (Eurogentec, Seraing, Belgium) following the manufacturer's instructions. The expression of mRNA in cells expressing the various shRNAs was related to an internal reference gene (see below) and their relative expression was quantified by the 2^−ΔΔCT^ method compared with the control condition (Livak and Schmittgen, 2001). TATA-binding protein (*Tbp*) mRNA was used as internal reference gene for the experiments of quantitative PCR on the neurons or blood cells. Glyceraldehyde 3-phosphate dehydrogenase (*Gapdh*) mRNA was used as the reference gene for the experiments of stability and β-glucuronidase (*Gusβ*) mRNA was used as reference gene for qPCR experiments on lymphoblastoid cells. All primer sequences are listed in Table S1 and S2 with the exception of primers for mouse FBJ murine osteosarcoma viral oncogene homolog (*cFo*s) and superoxide dismutase 1 (*Sod1*) that we previously published (Melko et al., 2013; Bechara et al., 2009; Maurin et al., 2015).

### Western blot

Protein extracts and western blots were performed as previously described (Melko et al., 2013). Previously described primary antibodies against CYFIP1 were polyclonal rabbit antibody 1665 (1:1000) (Schenck et al., 2001). Anti-β-actin monoclonal antibody (Sigma, Clone AC-15, A5441 lot 061M4808) and anti-mTor polyclonal rabbit antibodies (Cell Signaling, 2972 lot 2) were used at 1:10,000 and 1:1000, respectively. Anti-S6 total (Cell Signaling, 22175 lot 3), anti P-S6 (Cell Signaling, 48585 lot 3) and anti-PI3K p110β (Merck-Millipore, 09-482 lot 2786961) are polyclonal rabbit antibodies and were used as per manufacturer's instructions.

### Luciferase assays

STEK cells and 14 DIV cultured cortical neurons, expressing or not FMRP (Bechara et al., 2009; Maurin et al., 2015) and expressing ShScr or Sh89, were seeded in 96-well plates (20,000 cells/well) and transfected with the pRLTK constructs (with or without a G-quadruplex RNA-forming structure on the 5′UTR of the *Renilla* luciferase) and the pGL2-control (expressing a firefly luciferase as transfection control). Lipofectamine 2000 (Invitrogen) was used for transfection experiments following the manufacturer's instructions with slight modifications, as follows: 60 ng of DNA were used per well with 0.5 μl Lipofectamine 2000. After 48 h, the medium was removed and cells were lysed in 70 μl of 1× lysis buffer (Promega). *Renilla* and firefly activities were measured with the Dual-Glo luciferase assay (Promega) using a Glomax 96-well plate luminometer (Promega).

### Mouse dendritic spine analysis

Stereotaxic injections in wild-type and *Fmr1*-KO (strain C57/BL6) mice, histology and image analysis were performed as previously described (Daroles et al., 2015).

### Statistical analyses

All statistical analyses and graphs were realized using the GraphPad Prism Version 6.0e (GraphPad). The Mann-Whitney test was used to compare levels of mRNA or proteins measured by RT-qPCR or western blot, respectively. ANOVA followed by Newman-Keuls test for pairwise analysis was used to compare *Drosophila* NMJ length. ANOVA with Tukey's correction for multiple comparisons was used to compare the effect of both FMRP and CYFIP1 on G-quadruplex-dependent translation and on the level of pS6/S6. ANOVA with Dunnett's correction for multiple comparisons was used to compare the levels of the mRNAs of the WRC members in patients carrying deletion of the 15q11.2 chromosomal region with normal individuals. Crawford and Howell test was used to compare the level of WRC members in the patient carrying 4× the 15q11.2 chromosomal region with normal individuals. Two-way ANOVA was used to compare the morphology of dendritic spines in the different genotypes. Repeated-measures ANOVA with two factors was used to compare the dendritic arborization in the different genotypes.
